# Non-Invasive Prediction of Microvessel Density in Pediatric Hepatoblastoma Using Contrast-Enhanced Ultrasound Quantitative Parameters

**DOI:** 10.3390/diagnostics15212819

**Published:** 2025-11-06

**Authors:** Yazi You, Lirong Zhu, Hongli Zhai, Yuxin Tang, Jingyu Chen, Yi Tang

**Affiliations:** Department of Ultrasound, Children’s Hospital of Chongqing Medical University, National Clinical Research Center for Child Health and Disorders, Ministry of Education Key Laboratory of Child Development and Disorders, China International Science and Technology Cooperation Base of Child Development and Critical Disorders, Key Laboratory of Children’s Vital Organ Development and Diseases of Chongqing Health Commission, Chongqing 400014, China; youyazia@163.com (Y.Y.); masterzlr@163.com (L.Z.); zhl200208@163.com (H.Z.); 18323158273@163.com (Y.T.); cjy419103@163.com (J.C.)

**Keywords:** children, hepatoblastoma, ultrasonography, time–intensity curve, microvessel density

## Abstract

**Background**: The purpose of this study was to examine the relationship between qualitative characteristics and quantitative parameters from contrast-enhanced ultrasound (CEUS) and microvessel density (MVD) in hepatoblastoma (HB), as well as to investigate whether CEUS could be utilized as a non-invasive method for predicting HB progression. **Methods**: This retrospective analysis was carried out in one medical center and included 34 children with histopathologically confirmed HB. Both grayscale ultrasound and CEUS results were reviewed. Lesions were evaluated using time–intensity curve (TIC) analysis software to extract quantitative parameters. Postoperative tissue specimens were stained with CD34 immunohistochemistry, and MVD was quantified as the reference standard. Statistical analyses were conducted to assess the correlation between CEUS findings and MVD. **Results:** Lesions were separated into high (*n* = 21, 61.76%; MVD ≥ 41) and low (*n* = 13, 38.24%; MVD < 41) MVD groups, using the median microvessel density of 41 vessels per high-power field (HPF) as the cutoff. High MVD lesions exhibited a significantly higher incidence of penetrating vessels compared with low MVD lesions (*p* < 0.05). Elevated MVD levels were significantly associated with increased Adler-grade blood flow (*p* < 0.05). Both TIC-derived and relative quantitative parameters exhibited significant intergroup differences. Among the relative parameters, the relative wash-out rate (rWoR) was significantly higher in the low MVD group (*p* < 0.05). Moreover, the Receiver Operating Characteristic (ROC) curve analysis indicated that an rWoR threshold of ≥1.36 could serve as a predictor for low MVD, resulting in 76.9% sensitivity and 81.0% specificity (AUC = 0.802; 95% CI: 0.634–0.970; *p* = 0.003). **Conclusions**: CEUS revealed an association with MVD, supporting its potential as a non-invasive tool to characterize tumor vascularity.

## 1. Introduction

Hepatoblastoma (HB) is recognized as the predominant malignant solid liver tumor in pediatric patients [1], with vascular-dependent characteristics. Tumor angiogenesis is key to sustaining cancer cell survival, driving progression, and promoting metastasis.

Microvessel density (MVD), a widely used histopathological marker of angiogenesis, reflects the degree of tumor microvascular proliferation [2]. In various solid tumors, high MVD has been consistently linked to aggressive biological behavior, therapeutic resistance, and unfavorable survival outcomes [3]. In HB, angiogenesis enhances tumor vascularization, thereby promoting rapid tumor growth, aggressiveness, and metastatic potential [4,5]. Quantitative analyses have further shown that stratification by the median MVD can identify prognostic groups, with higher MVD associated with significantly poorer overall survival [6]. Anti-angiogenic therapy has also been considered a promising strategy, particularly in combination with individualized chemotherapy [7].Therefore, these findings suggest that MVD may serve as a clinically relevant biomarker for prognostic assessment in pediatric HB.

However, MVD assessment relies on histological specimens, which are invasive, subject to sampling error, unable to fully capture intratumoral heterogeneity, and unsuitable for longitudinal monitoring in children. These limitations highlight the need for reproducible, non-invasive approaches to evaluate tumor vascularity. Contrast-enhanced ultrasound (CEUS) with microbubble agents, such as SonoVue, enables real-time visualization of hepatic microcirculation [8]. In 2016, the U.S. Food and Drug Administration (FDA) approved SonoVue for pediatric liver imaging, providing both theoretical and technical support for its wider clinical application in children, including those with HB [9,10,11]. In adult solid tumors, quantitative CEUS parameters derived from time–intensity curve (TIC) analysis have shown significant correlations with histological MVD [12,13].

To date, no study has examined the association between CEUS parameters and MVD in pediatric HB. The present study therefore aimed to explore this relationship and to evaluate whether CEUS could serve as a non-invasive surrogate for histological vascular assessment in this population, in line with the increasing adoption of CEUS for pediatric focal liver lesions as supported by international guidelines [14].

## 2. Materials and Methods

### 2.1. Study Design

This study was conducted as a retrospective exploratory pilot investigation. Given the rarity of pediatric HB and the absence of prior work directly correlating CEUS parameters with histological MVD, the cohort size was determined by the availability of eligible patients during the study period. A post hoc power analysis based on the observed AUC of 0.802 for rWoR indicated that a sample size of 34 provides approximately 80% power at an alpha of 0.05, supporting the findings as sufficiently powered for this pilot investigation.

### 2.2. General Information

The Ethics Committee of Chongqing Children’s Hospital, Chongqing Medical University, approved this study (Approval No. 34/2024 Ethics Committee [Research]) in accordance with the Declaration of Helsinki issued by the World Medical Association. The children’ parents or legal guardians provided informed consent. Data from HB patients who underwent CEUS prior to surgery at our institution during the period of May 2019 to December 2024 were retrospectively analyzed in this study.

Inclusion criteria were as follows: (i) age < 18 years; (ii) histologically confirmed HB; (iii) both conventional ultrasound and CEUS performed before surgery; (iv) availability of complete resection specimens for immunohistochemical staining and MVD quantification. Exclusion criteria were as follows: (i) incomplete clinical data; (ii) severe motion artifacts on CEUS that, despite software correction, prevented generation of complete TICs and calculation of quantitative parameters; (iii) patients with only biopsy specimens available; (iv) cases with failed CD34 immunohistochemical staining.

**Figure 1:**
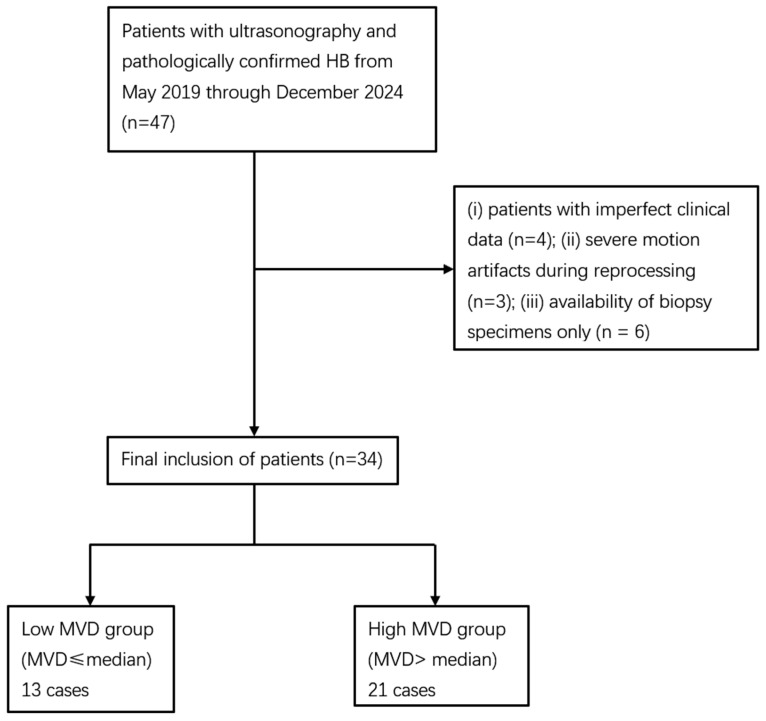
Flowchart of the study population. HB: hepatoblastoma; CEUS: contrast-enhanced ultrasound; MVD: microvessel density.

A total of 47 children were initially reviewed. Four cases were excluded due to incomplete clinical data, three because TIC analysis failed owing to severe motion artifacts, and six because only biopsy specimens were available, and no cases were excluded due to failed CD34 staining. Ultimately, 34 patients were included in the analysis. As no standardized guideline for MVD stratification has been established, patients in this cohort were divided according to the median MVD value (41 vessels per high-power field). This approach has been adopted in previous studies of HB [6] as well as in other tumor types [12]. Accordingly, they were categorized into a low MVD group (MVD < 41/HPF) and a high MVD group (MVD ≥ 41/HPF). The patient selection process is illustrated in Figure 1.

### 2.3. Methods

#### 2.3.1. Instrumentation

For the ultrasound examinations, an Acuson Sequoia unit (Siemens Medical Solutions USA, Mountain View, CA, USA) featuring a 1.0–4.0 MHz C251 convex array transducer was used. The contrast agent used in this study was SonoVue (sulphur hexafluoride microbubbles; Bracco Imaging, Milan, Italy), which is among the most widely used second-generation ultrasound contrast agents in pediatric practice. Other agents, such as Definity, Optison, and Sonazoid, are also clinically available [15,16]. SonoVue was administered at 0.03 mL/kg per patient according to FDA-recommended pediatric dosage guidelines, with a maximum volume not exceeding 2.4 mL per injection [14,17]. The excellent safety profile of SonoVue and other second-generation ultrasound contrast agents in the pediatric population is well-established [16].

#### 2.3.2. Examination Method

The examinee was placed in the supine position with the examination site fully exposed. Standard ultrasound was first performed to evaluate the lesion, documenting its dimensions, number, margin, calcification, and echogenic features. The background liver condition was also assessed using grayscale ultrasound combined with clinical records. Subsequently, the intralesional vascular signal was assessed via Color Doppler Flow Imaging (CDFI), resulting in a four-tiered classification of its vascularity according to Adler’s criteria [18]: grade 0, no blood flow signals; grade I, a small amount of blood flow, with 1~2 short rods or dots of blood flow; grade II, a moderate vascular signal characterized by several small vessels or a single vessel surpassing the lesion’s radius; grade III, a rich perfusion pattern, showing over four vessels or complex vascular networks interwoven into a reticular structure with mixed-color flow. Subsequently, based on conventional B-mode ultrasound findings, the axial or longitudinal plane displaying the maximum diameter of the lesion was selected as the acquisition plane for CEUS.

This plane was required to include both the lesion and the adjacent normal liver parenchyma to allow comparative evaluation of perfusion characteristics. Following plane selection, the contrast medium was administered intravenously via the elbow vein. The video capture function was activated simultaneously to monitor perfusion in both the lesion and adjacent normal liver tissue in real time. Throughout the 3–4-min imaging window, echo intensity variations were recorded and stored for analysis. (i) The enhancement uniformity was categorized as uniform, defined as homogeneous and evenly distributed intralesional perfusion without regional variation, or non-uniform, defined as heterogeneous enhancement with irregular hypo-enhancing areas, including non-enhancing regions that persisted through the arterial and portal venous phases. (ii) The enhancement pattern was classified as centripetal, when enhancement began at the periphery and progressed toward the center or centrifugal when enhancement began centrally and extended toward the periphery. (iii) Penetrating vessels were operationally defined as thin, linear, or branching enhancing structures originating from adjacent hepatic branches and extending from the tumor margin into the lesion during the arterial phase [19,20,21]. Each ultrasound assessment was conducted by a sonographer boasting 20 years of thorough expertise.

#### 2.3.3. Image Assessment

**Figure 2:**
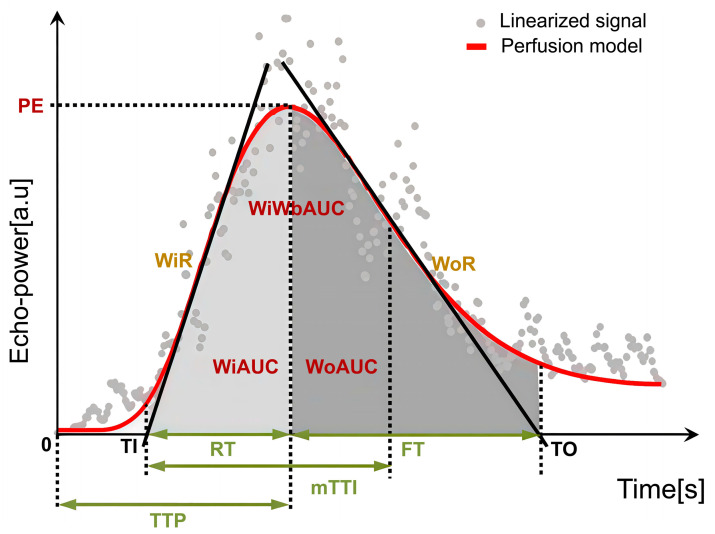
Schematic illustration of CEUS quantitative perfusion parameters.

Regions of interest (ROIs) were manually delineated on CEUS images using dedicated analysis software. For tumor ROIs, representative solid areas were selected while carefully avoiding non-enhancing anechoic regions corresponding to necrosis, strongly echogenic foci with posterior acoustic shadowing indicative of calcification, or large intra-tumoral vessels. A control ROI was placed in adjacent normal liver parenchyma at the same depth (same skin-to-ROI center distance) to minimize the influence of acoustic attenuation. All ROIs were determined by a single radiologist with more than 2 years of experience in pediatric CEUS, who was blinded to the pathological results. To reduce variability, circular ROIs with a fixed diameter of 10 mm were used across cases [22,23]. Respiratory motion artifacts were corrected using motion compensation algorithms. Time–intensity curve (TIC) analysis was then performed, and perfusion parameters were calculated as ratios between tumor and parenchyma to reduce variability related to ROI depth. Accordingly, the following relative quantitative parameters were obtained: relative image maximum (rImax), relative time to peak (rTTP), relative rise time (rRT), relative fall time (rFT), relative fall half time (rFHT), relative mean transit time (rmTT), relative area under the curve (rAUC), relative area under the curve (rWiAUC), relative wash-in area under the curve (rWoAUC), relative wash-in rate (rWiR), and relative wash-out rate (rWoR) in Table 1 and Figure 2. All CEUS parameters were reported with their respective units (s, %, or %/s). Tumor-to-liver ratios were additionally calculated as semi-quantitative parameters and reported without units.

#### 2.3.4. MVD Analysis

Immunohistochemical staining was performed on formalin-fixed, paraffin-embedded sections using a rabbit polyclonal antibody against CD34 (ZenBio, Chengdu, China; dilution 1:100). Antigen retrieval was performed using standard heat-induced epitope retrieval according to the manufacturer’s instructions. The primary antibody was incubated for 15 min, followed by incubation with a horseradish peroxidase–conjugated goat anti-mouse/rabbit IgG polymer III (Maixin, Fuzhou, China) for 30 min. Staining was visualized with a DAB kit (ZSGB-BIO, Beijing, China), and sections were counterstained with hematoxylin. Endothelial cells in non-tumoral vessels within the same sections served as the internal positive reference, and omission of the primary antibody served as the negative control.

Vascular “hot spots” were localized by initially scanning the entire slide under a 40× light microscope, following Weidner’s criteria [24], a well-established approach for MVD quantification in oncologic pathology [25]. Three areas exhibiting high vascular density were selected, and subsequently, the number of positively stained vessels within these hot spots was counted using a 200× light microscope. Neoplastic tumor blood vessels were identified as endothelial cells or cell clusters within the tumor tissue that stained brown or tan, distinctly separate from both tumor cells and neighboring microvessels. A single microvessel was enumerated at each point, and the MVD was determined as the mean of three such measurements. Each pathological interpretation was performed by a pathologist with 15 years of dedicated experience at our institution, who was blinded to patient sonographic information.

#### 2.3.5. Statistical Methods

The entirety of statistical analyses was performed using the SPSS software package (version 25.0, IBM Corporation, Armonk, NY, USA). The distribution of continuous variables was assessed using the Shapiro–Wilk test. Normally distributed data were expressed as mean ± SD and compared using the independent-samples *t*-test, while non-normally distributed data were expressed as median (IQR) and compared using the Mann–Whitney U test. Categorical variables were presented as counts and percentages, and group differences were assessed with the chi-square test. To evaluate the ability of CEUS quantitative parameters to differentiate varying degrees of MVD, receiver operating characteristic (ROC) curve analysis was performed, and the area under the curve (AUC), sensitivity, and specificity were calculated. Given the relatively small sample size and the exploratory nature of this study, no formal adjustment for multiple comparisons was applied. Statistical significance was defined as a two-sided *p* < 0.05.

## 3. Results

### 3.1. Patient Information and Safety

A total of 34 children with HB were included in the study. According to the median MVD value (41 vessels/HPF), 13 patients were classified into the low MVD group and 21 into the high MVD group. Baseline clinical characteristics, including demographic data, height, body weight, body mass index (BMI), serum alpha-fetoprotein (AFP), and liver function indices, are summarized in Table 2. Most patients presented with elevated AFP and increased transaminases, including alanine aminotransferase (ALT) and aspartate aminotransferase (AST), whereas albumin and total bilirubin were largely within normal ranges. BMI values were within the normal range for age, and all patients had a normal background liver without evidence of fatty infiltration, chronic hepatitis, or cirrhosis. No significant differences were observed between the high and low MVD groups. Age showed no significant association with CEUS parameters (*p* > 0.05), and subgroup analyses are provided in Appendix A Table A1.

For safety monitoring, key physiological indicators, including blood pressure, pulse rate, and oxygen levels, were recorded prior to, throughout, and half an hour following the ultrasound procedure. All children maintained stable vital signs—including heart rate, respiratory rate, oxygen saturation, and blood pressure—throughout the CEUS examination. No adverse events such as nausea, vomiting, fever, or rash were reported.

### 3.2. Ultrasound Features of HB Lesions: High and Low MVD Groups

The two groups showed no significant differences in dimensions, number, echogenicity, margin, or calcification (*p* > 0.05). Nonetheless, a statistically significant variation in CDFI blood-flow grades was observed between groups (*p* < 0.05), as shown in Table 3. In the low-MVD group, 4/13 (30.77%) tumors presented Adler grade 0–I, while 9/13 (69.23%) showed Adler grade II–III. In contrast, all 21/21 (100%) tumors in the high-MVD group demonstrated Adler grade II–III.

### 3.3. Qualitative CEUS Performance of HB Lesions: High and Low MVD Groups

**Figure 3:**
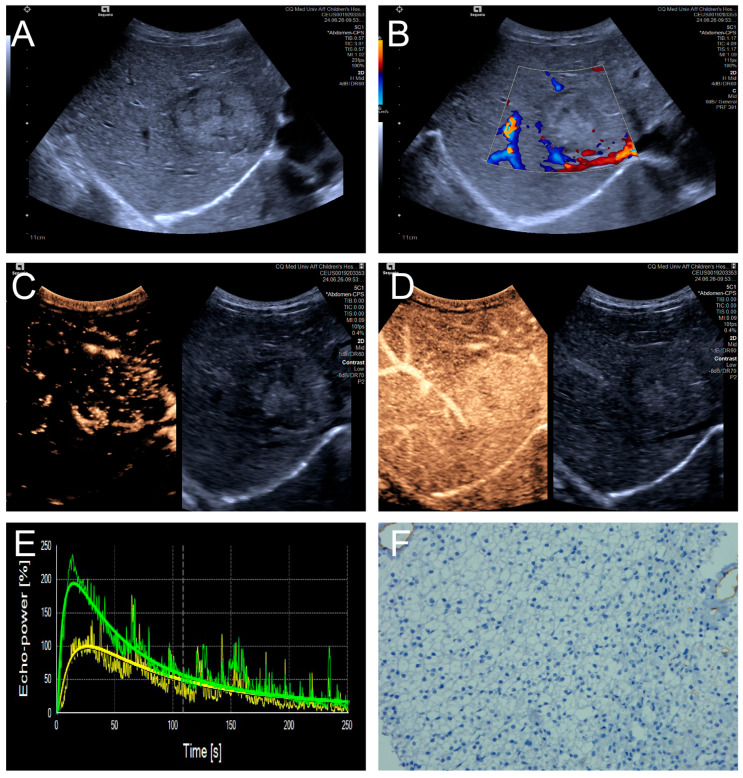
(**A**–**F**) A male patient aged 4 years and 4 months with HB and low MVD. (**A**) The imaging revealed a well-defined mass with clear margins and hyperechoic echogenicity in the right hepatic lobe; (**B**) The tumor’s color Doppler flow imaging revealed distinct blood flow patterns and Adler grade I blood flow; (**C**,**D**) The CEUS exhibited uniform internal enhancement from periphery to center without any peripheral penetrating vessels; (**E**) TIC curves showed contrast enhancement of the lesion (green) and perfusion of the adjacent liver parenchyma (yellow). Jagged lines indicated raw signal data, and smooth lines indicated the fitted curves; (**F**) The biopsy specimen’s cytological immunohistochemical staining revealed few microvessels (CD34 staining × 200), and the MVD measured 21 lines/HPF.

**Figure 4:**
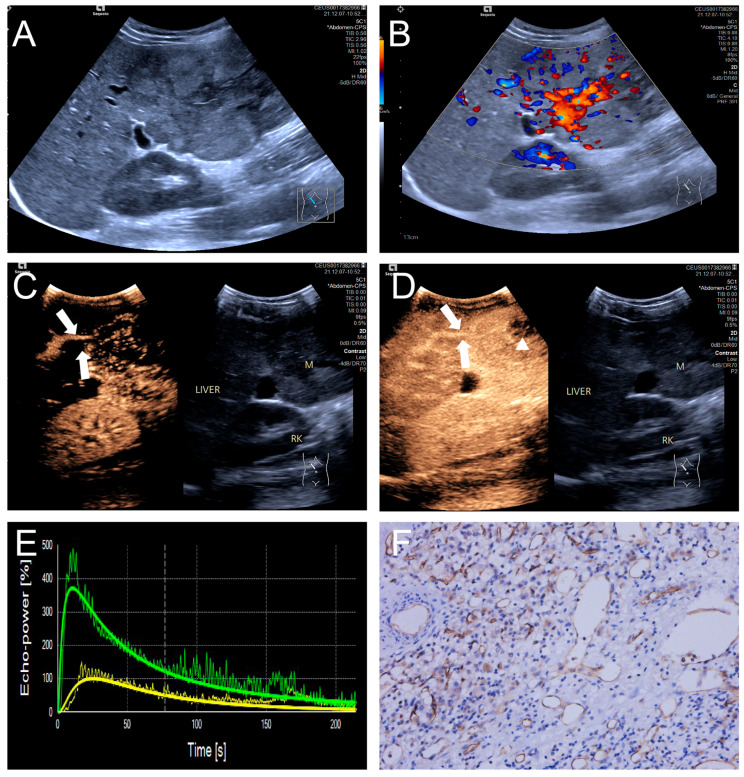
(**A**–**F**) A male patient aged 10 years and 6 months with HB and high MVD. (**A**) The imaging revealed a lesion with unclear margins and hyperechoic echogenicity in the liver’s left lobe; (**B**) During the enhancement phase, CDFI revealed abundant vascular signals within the tumor tissue, classified as grade III by Adler; (**C**,**D**) The CEUS exhibited non-uniform enhancement progressing from periphery to center, with non-enhancing regions (triangles) within the lesion and peripheral penetrating vessels (arrows); (**E**) TIC curves showed contrast enhancement of the lesion (green) and perfusion of the adjacent liver parenchyma (yellow). Jagged lines indicated raw signal data, and smooth lines indicated the fitted curves; (**F**) The biopsy specimen’s cytological immunohistochemical staining revealed dense microvessels (CD34 staining × 200), and the MVD measured 42 lines/HPF.

Penetrating vessels were significantly more frequent in the high MVD group (*p* < 0.05). In contrast, no significant differences were observed between the two groups regarding enhancement uniformity or enhancement order (*p* > 0.05). Representative CEUS features are illustrated in Figure 3 and Figure 4, and the comparative results are summarized in Table 4.

### 3.4. CEUS Perfusion Parameters of HB Lesion Tissues: High and Low MVD Groups

The low MVD group exhibited significant differences in TTP (s), RT (s), FHT (s), Imax (%), and WoR (%) when compared to the adjacent healthy hepatic tissue (*p* < 0.05). Compared to adjacent non-tumorous hepatic tissue, high MVD lesions showed distinct characteristics in TTP (s), RT (s), and FHT (s) (*p* < 0.05); the other evaluated parameters did not exhibit meaningful variations between these tissue types (*p* > 0.05), as listed in Table 5. To minimize variability related to ROI depth, relative parameters were calculated as tumor-to-liver ratios for intergroup comparisons. The analysis indicated that rImax, rTTP, rRT, rFHT, rAUC, rWoAUC, and rWoR showed significant intergroup differences (*p* < 0.05), while no such significance was found for rFT, rmTT, rWiAUC, or rWiR, as detailed in Table 6.

### 3.5. ROC Curve and Box Plot Analysis of Relative CEUS Parameters of HB Lesion Tissues: High and Low MVD Groups

**Figure 5:**
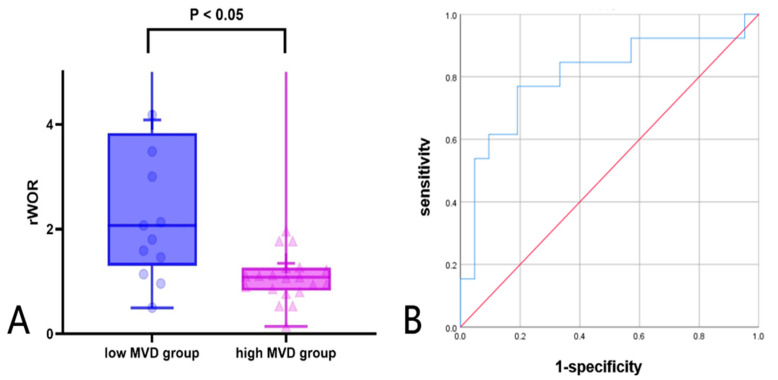
(**A**,**B**) Diagnostic performance of rWOR in high and low MVD groups (**A**) box plot; (**B**) ROC curve: receiver operating characteristic curve.

Further ROC analysis was conducted to evaluate the diagnostic performance of CEUS relative parameters. Among these semi-quantitative parameters, rWoR showed the best performance in differentiating high and low MVD groups. The AUC of rWoR was 0.802, with a cutoff value of 1.36, a diagnostic sensitivity of 76.9%, and a specificity of 81.0% in Figure 5A and Table 7. To further visualize the distribution of rWoR across different MVD groups, a box plot was constructed, which showed that the rWoR values in the low MVD group were significantly higher than those in the high MVD group (*p* < 0.05) in Figure 5B.

## 4. Discussion

Hepatoblastoma, a common pediatric malignancy, accounts for approximately 90% of hepatic malignancies below five years old [26,27]. Currently, grayscale ultrasound struggles to identify microscopic blood flow and liquefied necrosis tissue. Contrast-Enhanced Computed Tomography (CECT) and Magnetic Resonance Imaging (MRI) are alternative imaging methods that can offer additional information about the surrounding tissues. Nevertheless, CT exposes children to potentially harmful radiation, and MRI is limited by its lengthy procedure and poor patient compliance. In contrast, CEUS avoids radiation exposure and permits continuous real-time visualization of microvasculature, making it uniquely advantageous for children. This aligns with the WFUMB-EFSUMB guidelines, which highlight CEUS as a valuable, real-time, and safe modality for characterizing focal liver lesions [28].

MVD serves as a crucial indicator for evaluating tissue angiogenesis, effectively indicating the proliferation and invasiveness of tumor cells. This retrospective analysis explored the association of CEUS with MVD expression in HB. The findings indicated that the low MVD group showed a lower incidence of penetrating vessels while also exhibiting more quantitative parameters that differed significantly from the surrounding normal parenchyma. In addition, the high and low MVD groups differed in rImax, rTTP, rRT, rFHT, rAUC, rWoAUC, and rWoR. Specifically, rWoR was significantly higher in the low MVD group than in the high MVD group, suggesting its potential advantage in reflecting generation. Further analysis revealed that rWoR ≥1.36 could be a valid parameter for predicting low MVD expression in HB preoperatively.

The growth, invasion, and metastasis of cancer cells are closely dependent on their microvascular supply [29,30]. As a histological marker of angiogenesis, MVD reflects tissue microvasculature and has been correlated with tumor growth, recurrence, and prognosis in multiple malignancies. For example, Goyal et al. [31] reported that breast tumors with higher MVD exhibited greater proliferative capacity, while Zvrko [32] showed that elevated MVD in laryngeal cancer was associated with recurrence, advanced stage, and lymph node metastasis. Collectively, these findings indicate that high MVD generally corresponds to more aggressive tumor biology and poorer prognosis. In HB, patient stratification by the median MVD has also demonstrated prognostic differences [6]. So we adopted the cohort median as the cutoff in the present study to assess its association with CEUS parameters.

Assessment of MVD currently relies on tumor tissues collected via surgical excision or needle biopsy. However, the invasiveness and heavy reliance on operator expertise of these techniques limit their broader clinical application. Therefore, it is crucial to adopt a non-invasive imaging method that effectively and rapidly predicts MVD counts. CEUS is becoming a recognized and prospective way to assess MVD in hepatic neoplasms. Faccia et al. [33] reported that CEUS quantitatively evaluates capillary flow and reliably supervises antiangiogenic therapy outcomes, with a diagnostic accuracy comparable to CT and MRI. Also, Mostafa et al. [11] observed that CEUS, by imaging tumor vasculature before surgery, facilitated early diagnosis, personalized therapy, and medical outcomes.

All tumors in the high MVD group showed Adler grade II–III blood flow, and 9 of 13 tumors in the low MVD group also demonstrated grade II–III signals (69.23%). Adler grading is semi-quantitative and dependent on machine parameters and acquisition settings, and Doppler is preferentially sensitive to larger-caliber vessels or higher-velocity flow while relatively insensitive to slow capillary perfusion. Consistent with these properties, the frequent grade II–III signals in the low MVD group indicate that Doppler is not a reliable surrogate for capillary-level perfusion, despite a group-level association with MVD. By contrast, CEUS provides not only quantitative parameters from TIC analysis but also information on the dynamic characteristics of microvascular perfusion at the capillary level, thereby offering incremental value beyond Doppler, which is limited to depicting larger, faster-flowing vessels [34].

Currently, no study has been found to apply CEUS to predict MVD counts in pediatric HB. Using CEUS, this study offered a real-time, dynamic visualization of microcirculatory perfusion within lesions. A significant association was observed between the presence of penetrating vessels and MVD. In the high MVD group, peripheral penetrating vessels were frequently observed. This observation is consistent with previous findings. Li et al. [35] reported that the peripheral regions of tumors typically have significantly higher MVD than the central areas, and the denser peripheral vasculature may facilitate the formation of penetrating vessels [36]. Tumor morphology may change before angiogenesis commences, and grayscale sonography cannot discern the extent of tissue infiltration. By contrast, microbubble agents enable the visualization of blood flow, specifically revealing penetrating and irregular vessels [37].

To minimize visual bias and subjectivity, quantitative analysis software was applied to objectively characterize perfusion distribution in the form of quantitative parameters [38]. Differences between high MVD lesions and the surrounding liver parenchyma were mainly reflected in TTP (s), RT (s), and FHT (s). In contrast, low MVD lesions demonstrated additional significant discrepancies, including Imax (%) and WoR (%). Imax (%) reflects the vascularity and blood inflow of the lesion, being closely related to its perfusion status. In the low MVD group, Imax (%) was significantly higher than that of the surrounding parenchyma, indicating stronger arterial enhancement. Such a pattern may be attributed to the relatively preserved vascular integrity in low MVD tumors, which facilitates microbubble inflow, increases the effective intravascular blood pool volume, and generates more homogeneous peak enhancement, consistent with the findings of Li et al. [39]. WoR (%) represents the washout rate of the contrast agent [34]. In low MVD lesions, WoR (%) was also significantly higher than that of the background liver, suggesting more rapid clearance of microbubbles. This likely reflects that, although relatively preserved vascular integrity allows higher Imax through rapid arterial inflow, the sparse vascular network limits perfusion sustainability, leading to insufficient retention and consequently faster washout.

To further reduce potential confounding, we compared relative parameters between the high and low MVD groups. Significant differences were observed in rImax, rTTP, rRT, rFHT, rAUC, rWOAUC, and rWoR. Specifically, the high MVD group exhibited elevated rTTP, rRT, and rFHT, indicating delayed peak enhancement and prolonged washout. This may be explained by the fact that, although containing more neovessels, their abnormal, tortuous structure and high permeability limit effective vascular volume and perfusion efficiency, leading to slower inflow and delayed clearance [40]. In contrast, the low MVD group showed higher rImax, rAUC, rWOAUC, and rWoR, reflecting stronger arterial enhancement and greater overall perfusion. With fewer but relatively intact vessels, these lesions might achieve more efficient perfusion, resulting in rapid inflow, accelerated clearance, and a larger effective blood volume.

Among these parameters, rWoR yielded the largest area under the ROC curve, indicating superior diagnostic performance in distinguishing between high and low MVD. rWoR expresses the clearance rate as the ratio of lesion to background liver, providing a more objective measure of their relative difference [41]. In low MVD lesions, the limited number of vessels shortened intratumoral transit, leading to more rapid enhancement decline compared with the background liver. This amplified the lesion-to-background difference, leading to elevated rWoR. In contrast, high MVD tumors, with abundant neovessels, arteriovenous shunts, and increased permeability, also showed accelerated clearance. However, the surrounding liver parenchyma, with its rich perfusion, cleared at a similarly rapid pace, thereby attenuating the relative difference and resulting in lower rWoR [34,42].

Therefore, the quantitative CEUS parameters were associated with intratumoral MVD. On this basis, rWoR may be further explored as a non-invasive indicator to assess intratumoral vascularity, thereby potentially reducing reliance on invasive biopsy and enabling longitudinal monitoring of microvascular changes for evaluating responses to anti-angiogenic therapy. Moreover, since higher MVD has been linked to more aggressive tumor biology and poorer prognosis, CEUS perfusion parameters may also provide supportive information for risk stratification. These potential implications remain exploratory and warrant further investigation in future clinical research.

## 5. Limitations

This study has several limitations. First, this was a retrospective, single-center pilot study with a limited sample size and no formal sample size calculation. Larger, prospective multicenter studies are required to validate these findings. Second, the median value of MVD was used for grouping, following the previous literature, but standardized thresholds should be established in future work. Third, interobserver variability in CEUS quantification was not assessed. The ROI analysis was performed by a single reader, which may introduce bias and limits the generalizability of the absolute parameter values. Future studies assessing reproducibility across observers are needed to confirm the reliability and generalizability of CEUS quantification [38]. Finally, this study did not include direct comparison with MRI perfusion. CEUS was evaluated as an independent modality with potential utility for real-time, radiation-free assessment of tumor vascularization. Future studies integrating CEUS with MRI or other modalities would be valuable for broader clinical validation.

## 6. Conclusions

In conclusion, as a non-invasive approach to vascular assessment, CEUS showed significant differences in TIC-derived parameters and relative parameters between high and low MVD groups in pediatric HB. Among the assessed parameters, rWoR was most helpful for group discrimination.

## Figures and Tables

**Table 1:** Quantitative parameter definitions.

Parameter Abbreviation	Full Parameter Name	Definition	Unit
Imax	Image Maximum	Maximum relative echo intensity within the ROI	%
TTP	Time to Peak	Time interval from contrast arrival to maximum intensity	s
RT	Rise Time	Time interval from contrast arrival to the onset of the peak	s
FT	Fall Time	Time interval from maximum intensity to the return to baseline	s
FHT	Fall Half Time	Time required for the signal to decrease to 50% of the maximum intensity during wash-out	s
mTT	Mean Transit Time	Mean transit time of microbubbles within the ROI	s
AUC	Area Under the Curve	Total area under the time–intensity curve	%/s
WiAUC	Wash-in Area Under the Curve	Area under the curve during the wash-in phase	%/s
WoAUC	Wash-out Area Under the Curve	Area under the curve during the wash-out phase	%/s
WiR	Wash-in Rate	Slope of the ascending portion of the curve during contrast wash-in	%
WoR	Wash-out Rate	Slope of the descending portion of the curve during contrast wash-out	%

ROI: region of interest.

**Table 2:** Baseline demographic and biochemical characteristics of pediatric HB patients stratified by MVD status.

Variable	Overall (*n* = 34)	Low MVD Group (*n* = 13)	High MVD Group (*n* = 21)	*p* Value
Age (years)	2.00 (0.96, 3.52)	1.30 (0.71, 3.25)	2.00 (1.38, 4.42)	0.152
Sex (male/female)	19/15	6/7	13/8	0.484
Height (cm)	2.00 (0.96, 3.52)	74.5 (73.0, 97.5)	84.0 (75.5, 101.9)	0.104
Weight (kg)	2.00 (0.96, 3.52)	8.75 (8.00, 13.13)	12.00 (9.75, 15.15)	0.175
BMI (kg/m^2^)	15.60 ± 1.81	10.91 ± 3.75	20.83 ± 8.14	0.103
ALT (U/L)	221.55 (141.50, 345.13)	227.00 (146.53, 464.25)	218.00 (130.45, 320.00)	0.671
AST (U/L)	356.85 (196.50, 586.18)	446.05 (216.33, 578.50)	310.00 (217.50, 598.35)	0.671
Tbil (µmol/L)	14.00 (11.65, 20.90)	14.00 (10.60, 17.83)	13.00 (112.30, 21.85)	0.326
ALB (g/L)	48.35 ± 3.13	48.28 ± 3.64	48.22 ± 2.88	0.709
AFP (ng/mL)	177,360.00 (15,996.75, 563,285.50)	58,395.00 (945.00, 58,395.00)	228,124.00 (33,083.00, 588,438.00)	0.365

BMI: body mass index; ALT: alanine aminotransferase; AST: aspartate aminotransferase; Tbil: total bilirubin; ALB: albumin; AFP: alpha-fetoprotein; MVD: microvessel density.

**Table 3:** Ultrasound features of HB lesions: high and Low MVD groups.

Parameter	Low MVD Group (*n* = 13)	High MVD Group (*n* = 21)	*p* Value
Size (cm)	9.57 ± 3.82	10.62 ± 3.90	0.206
Number			0.513
Single	13 (100%)	19 (90.48%)	
Multiple	0 (0%)	2 (9.52%)	
Echogenicity			1.000
Hyperechoic	10 (76.92%)	16 (76.19%)	
Hypoechoic	3 (23.08%)	5 (23.81%)	
Margin			1.000
Clear	10 (76.92%)	16 (76.19%)	
Unclear	3 (23.08%)	5 (23.81%)	
Calcification			0.481
Absent	8 (61.54%)	9 (42.86%)	
Present	5 (38.46%)	12 (57.14%)	
Alder Grading			0.005
Grade 0–I	4 (30.77%)	0 (0%)	
Grade II–III	9 (69.23%)	21 (100%)	

MVD: microvessel density.

**Table 4:** Qualitative CEUS performance of HB lesions: high and low MVD groups.

Parameter	Low MVD Group (*n* = 13)	High MVD Group (*n* = 21)	*p* Value
Uniformity			0.076
Uniform	9 (69.23%)	7 (33.33%)	
Non-uniform	4 (30.77%)	14 (66.67%)	
Enhancement Order			0.724
Centripetal	4 (30.77%)	8 (38.10%)	
Centrifugal	9 (69.23%)	13 (61.90%)	
Penetrating Vessels			0.032
Absent	10 (76.92%)	7 (33.33%)	
Present	3 (23.08%)	14 (66.67%)	

MVD: microvessel density.

**Table 5:** Quantitative CEUS parameters of HB lesion tissues: high and low MVD groups.

Parameter	Lesion	Normal Tissue	*p* Value
Low MVD Group (*n* = 13)			
Imax (%)	185.08 (116.93, 267.45)	100.00 (100.00, 100.00)	0.001
TTP (s)	26.11 ± 7.17	12.62 ± 4.34	0.001
RT (s)	22.94 ± 7.52	11.31 ± 3.80	0.001
FT (s)	51.54 (37.25, 99.62)	59.20 (41.80, 113.76)	0.511
FHT (s)	23.92 (17.29, 42.88)	36.99 (31.19, 59.73)	0.010
mTT (s)	42.87 (32.74, 111.33)	65.49 (52.29, 154.77)	0.057
AUC (%/s)	67,996.01 (30,887.42, 80,280.39)	53497.74 (27,311.59, 74,955.70)	0.650
WiAUC (%/s)	921.86 (340.97, 3689.97)	2489.07 (749.23, 3578.87)	0.311
WoAUC (%/s)	35,574.79 (17,945.04, 63,636.06)	23,798.84 (8395.34, 45,198.85)	0.186
WiR (%)	74.58 (36.18, 386.26)	124.03 (28.47, 163.30)	1.000
WoR (%)	594.81 (278.14, 1207.55)	343.69 (162.59, 462.03)	0.007
High MVD Group (*n* = 21)			
Imax (%)	107.96 (92.12, 124.82)	100.00 (100.00, 100.00)	0.158
TTP (s)	12.88 (9.93, 16.48)	19.08 (12.38, 24.08)	0.012
RT (s)	12.91 ± 4.74	12.91 ± 4.74	0.007
FT (s)	12.91 ± 4.74	12.91 ± 4.74	0.248
FHT (s)	34.54 ± 16.71	34.54 ± 16.71	0.013
mTT (s)	64.00 (41.34, 84.53)	87.46 (58.32, 161.32)	0.080
AUC (%/s)	35,046.53 (17,842.05, 76,627.77)	54,267.17 (28,049.96, 85,031.29)	0.359
WiAUC (%/s)	451.46 (191.58, 855.01)	799.22 (299.95, 1594.27)	0.204
WoAUC (%/s)	21,084.06 (10,086.15, 491,066.75)	23,419.92 (13,638.32, 51,956.02)	0.660
WiR (%)	42.90 (16.81, 74.29)	39.10 (19.52, 86.03)	0.772
WoR (%)	351.28 (189.64, 547.09)	454.32 (162.76, 481.57)	0.831

**Table 6:** Relative CEUS parameters of HB lesion tissues: high and low MVD groups.

Parameter	Low MVD Group (*n* = 13)	High MVD Group (*n* = 21)	*p* Value
rImax	1.85 (1.17, 2.67)	1.08 (0.92, 1.25)	0.014
rTTP	0.46 (0.31, 0.67)	0.62 (0.53, 0.96)	0.010
rRT	0.48 (0.33, 0.65)	0.62 (0.53, 0.98)	0.030
rFT	0.83 ± 0.30	0.94 ± 0.36	0.370
rFHT	0.56 ± 0.17	0.74 ± 0.26	0.023
rmTT	0.68 (0.56, 0.74)	0.68 (0.54, 0.97)	0.344
rAUC	1.12 (0.93, 1.42)	0.77 (0.66, 1.00)	0.027
rWiAUC	0.63 (0.17, 1.61)	0.68 (0.29, 1.50)	0.889
rWoAUC	1.24 (1.00, 2.58)	0.80 (0.72, 1.35)	0.024
rWiR	1.11 (0.46, 3.69)	0.89 (0.53, 1.40)	0.576
rWoR	2.07 (1.30, 3.83)	1.08 (0.83, 1.26)	0.003

Imax: image maximum; TTP: time to peak; RT: rise time; FT: fall time; FHT: fall half time; mTT: mean transit time; AUC: area under the curve; WiAUC: wash-in area under the curve; WoAUC: wash-out area under the curve; WiR: wash-in rate; WoR: wash-out rate; MVD: microvessel density.

**Table 7:** ROC curve analysis of relative CEUS parameters of HB lesion tissues: high and low MVD group.

Parameter	AUC	*p* Value	95%CI	Cut-Off Point	Sensitivity	Specificity
rWoR	0.802	0.003	0.634–0.970	1.36	0.769	0.810

AUC: area under the curve; rWoR: relative wash-out rate; CI: confidence interval.

## Data Availability

The original contributions presented in this study are included in the article/Appendix A. Further inquiries can be directed at the corresponding author.

## Abbreviations

The following abbreviations are used in this manuscript:

CEUS	Contrast-Enhanced Ultrasound
MVD	Microvessel Density
HB	Hepatoblastoma
TIC	Time–Intensity Curve
CDFI	Color Doppler Flow Imaging
ROC	Receiver Operating Characteristic
ROI	Region Of Interest
Imax	Image Maximum
TTP	Time to Peak
RT	Rise Time
FT	Fall Time
FHT	Fall Half Time
mTT	Mean Transit Time
AUC	Area Under The Curve
WiAUC	Wash-Out Area Under The Curve
WoAUC	Wash-In Area Under The Curve
WiR	Wash-In Rate
WoR	Wash-Out Rate
CT	Computed Tomography
MRI	Magnetic Resonance Imaging

## Appendix A

**Table A1:** Association between CEUS quantitative parameters and age subgroups in pediatric HB.

Quantitative Parameter	<3 Years (*n* = 22)	≥3 Years (*n* = 12)	*p* Value
Imax (%)	115.99 (92.36, 207.13)	121.91 (108.74, 205.71)	0.582
TTP (s)	11.97 ± 4.25	23.30 ± 8.53	0.783
RT (s)	10.91 ± 3.75	20.83 ± 8.14	0.792
FT (s)	62.35 (40.57, 102.73)	59.61 (49.89, 77.43)	0.958
FHT (s)	28.06 (18.20, 44.91)	27.97 (21.84, 33.39)	0.901
mTT (s)	58.23 (36.03, 97.33)	50.91 (41.78, 82.87)	0.790
AUC (%/s)	45,047.21 (25,833.10, 75,984.30)	44,040.51 (17,668.64, 82,750.17)	0.709
WiAUC (%/s)	759.82 (248.85, 1714.97)	421.88 (198.85, 779.55)	0.292
WoAUC (%/s)	26,214.10 (14,609.24, 52151.01)	26,417.45 (9708.47, 65,472.60)	0.736
WiR (%)	59.98 (29.00, 120.61)	40.95 (16.33, 59.14)	0.292
WoR (%)	467.45 (260.68, 732.62)	417.67 (189.96, 579.68)	0.683

Imax: image maximum; TTP: time to peak; RT: rise time; FT: fall time; FHT: fall half time; mTT: mean transit time; AUC: area under the curve; WiAUC: wash-in area under the curve; WoAUC: wash-out area under the curve; WiR: wash-in rate; WoR: wash-out rate.
